# Blistering—Particularly in Reference to Children

**Published:** 1875-07

**Authors:** E. T. Dorland


ART. II.—*Blistering—Particularly in Reference to Children.* By E. T. DORLAND, M. D. Read before the Erie County Medical Society, January 12, 1875, and referred to the BUFFALO MEDICAL AND SURGICAL JOURNAL for publication.

Entertaining the opinion that blistering has fallen into too great disrepute, especially in reference to its application to children, and that this prejudice is in some measure owing to a lack of understanding of the peculiarities of operation, and of the manner of applying blisters to young subjects, I propose to consume a few moments of your time in presenting my ideas in regard to this important subject. No new light is expected to be thrown upon the subject of the indications for blistering, but a hope is entertained that a little may be done in this effort toward clearing away some of the objections to the use of blisters, so that this potent therapeutic agent may not be charged with more evil than properly belongs to it. We will first enquire into the peculiarities of the operation of blisters on children, and lastly we will consider the manner of applying the same.

The first peculiarity attending the operation of blisters on the young subject is that they produce their effect in a shorter time than in the adult—this is a fact well known. In this respect there is a striking difference between blisters and most other remedies. Emetics and cathartics, for example, do not appear to act with any more rapidity on the child than on the adult. This fact of the more prompt action of this class of agents upon the child, although a simple one, is nevertheless one of much importance. It has a practical bearing not merely upon the mode of conducting the process of blistering in young subjects, but also upon the use of it in their various diseases. The second peculiarity is that the local inflammation produced by a blister is greater in the young subject than in the adult. The reason of this is obvious; in infancy the skin is more delicate in structure, has a greater vascularity and a higher degree of sensibility; all circumstances favoring the development of greater inflammation, the local impression accordingly made by a blister is not merely more rapid in the young subject but it is also more intense.

The third peculiarity is that in young subjects blisters are more apt to be followed by the injurious consequences of inflammation, such as ulceration, gangrene, and even death. These occurrences may and do take place also in the adult, but they are comparatively rare and only under very peculiar states of the system. In any child, however healthy, they may occur from the simple cause of the blister being left on too long; they are more likely to take place, however, in certain conditions of the system or of the skin itself; thus, for example, in cases where a child is greatly emaciated or the constitution broken down from various causes, the inflammation of a blister is apt to become unhealthy in its character and to be followed by injurious consequences; also when the skin is diseased unpleasant consequences are very apt to follow.

The fourth peculiarity is that the constitutional excitement produced by blisters is generally greater in young subjects than in adults—that this must necessarily be so is obvious. In all cases the general excitement must be in proportion to the degree of local irritation and the sensibility of the patient's system.

From the foregoing statements it is evident that blisters are much more powerful in their agency upon the young subject than upon the adult. They operate with more rapidity and cause a greater degree of local irritation and constitutional excitement. If such be case, it appears to me that some conclusions may be drawn of no inconsiderable practical importance. If blisters are more powerful in their action upon children than adults, then it would seem to follow that they may be rendered more efficient as a means of cure in their diseases, and such appears to me to be the fact. In all cases where their revulsive agency is required, and where they are properly applied, it has struck me that more decided benefit has resulted from their use in children than in adults, and that, too, under circumstances as nearly similar as they well could be. We all know that one of the greatest objections to a blister in the adult, sometimes at least, is the length of time which it takes to produce its effects; in a child this is in a great measure obviated, and we have in a blister not merely a powerful but a comparatively speedy counter irritant.

The opposers of blistering will tell us that we first subdue the severity of the disease by other and appropriate remedies, and when it is upon its decline, when in all probability the unassisted powers of nature would successfully perform the remainder of the task, a blister is applied; the patient gets well notwithstanding the additional pain thus inflicted, and the fortunate results of the case, which is really to be attributed to the measures previously employed, is said to be owing to the good effects of counter irritation, etc. Now all this may, no doubt, be true in some cases, but that it is so generally can hardly be admitted. It should be recollected that in the treatment of local inflammation blisters are only auxiliary remedies of themselves, and alone capable of doing but little, perhaps, and yet when co-operating with other agents, such as blood-letting, etc., really powerful and valuable. Every one knows that there are periods and conditions in the career of inflammatory complaints when bleeding and other reducing remedies have been carried to their fullest extent deemed advisable, and yet sufficient disease may remain, if not to destroy life, yet to render convalescence tedious or to lay the foundation of subsequent chronic disease. It is just under this condition of things that blisters come in with great effect, and frequently break up completely the remaining vestiges of disease, and in this way I look upon them as remedies acting with more power and efficiency in children even than in adults. From the fact of blisters being such powerful agents, and especially from the fact of their being so liable to be followed by dangerous consequences, more caution is required in their use in children than in adults. Important and valuable as they are, and may be made if properly used, their indiscriminate application cannot be too much reprobated. Just in proportion to the good they are capable of accomplishing under proper circumstances, is the evil which results from them if heedlessly or injudiciously resorted to. This fact should never be lost sight of when we are contemplating the use of blisters, especially upon very young children.

Lastly, we come to consider the manner of blistering. The mode of conducting the process of blistering in a young subject is a matter of great nicety, and should call for the utmost care on the part of the practitioner. As one of the principal cause of gangrene is leaving the blister on too long, this is a point which should be especially attended to. The directions given by many writers in reference to this are very indefinite and discordant. By way of illustration, I will quote some of them. Dr. Armstrong says from twelve to sixteen hours is generally sufficient for the application of the blister in adults, and half that period in children. Dr. Williams says to avoid gangrene in children, it is advisable never to allow the blister to remain on more than six hours. Dr. Dewees states that in children the blister is frequently found to have performed its duty in eight hours, and very often in six. Neligan directs that as a general rule in infants and young children, blisters should only be left on until redness of the surface is produced, when the application of a warm poultice to the part will cause vesication. The foregoing is a sample of the discrepancy of opinion in relation to a most important point of practice. The fact is that no positive rule can be laid down in relation to the precise time that a blister should be left on a young child. From the original differences in the sensibility of the skin in children, the period must necessarily vary, and therefore the effect produced is the only correct guide as to time. For this purpose the plaster should be raised often, and the state of the skin observed, and as soon as the surface appears uniformly red the plaster should be removed and a poultice applied. In most cases this will be followed by suitable vesication, while any injurious consequences will be averted. It was not my intention in this paper to go into the minutiæ of conducting the process of blistering, but there is a point or two more that I cannot help noticing. The first I will refer to is the habit I have observed with some of covering the blistering plaster with dry fly powder. Although intended to make the blister more potent, it frequently has a directly contrary effect, from the fact that the blister does not adhere so closely to the skin. Then, again, the dry powder is apt to adhere to the skin after the blister is removed, and in this way, in my opinion, stranguary is more likely to be produced. With regard to the dressing of a blister, always a matter of importance to the young subject, and frequently so to the adult, I have for many years practiced and greatly admire the plan recommended many years ago by Dr. D. Maclagan, of Scotland, which, I think, holds out many advantages. It is as follows: After leaving the blister on for a suitable time apply a poultice of bread and milk for two hours; after discharging the serum apply a thick layer of soft cotton wadding, with the undressed or woolly side next the skin; if in the course of a few hours this should become soaked with the serous discharge from the blister, so much of the cotton may be removed as can be done without disturbing the loose epidermis beneath, and the whole again covered with a dry layer of cotton. This is all the dressing that is generally requisite. The cotton is allowed to stick to the skin of the blistered part, and when a fresh layer of epidermis is formed, which takes place very readily, the old epidermis and cotton come off together, leaving a smooth, whole surface below. The advantages of the above mode, according to Dr. Maclagan, are first, that it renders the blister much less painful and annoying to the patients than when unguents are used. The tenderness, in fact, is comparatively so trifling, and the protection by the cotton so good, he says, that I have been enabled, without annoyance to the patient, to percuss freely and apply the stethoscope firmly over blistered parts which had been dressed for the first time only an hour or two previously. Secondly, blisters heal much more readily dressed in this way; and lastly, it is much more agreeable to persons of cleanly habits than the dressings with cerates.

To obtain the good and avoid the evils of blisters, it is evident that a nicer discrimination of the conditions of the system is necessary in the use of this class of agents in children than in adults. Long experience has established the fact that it is only under certain states of the system that blisters can be used with any prospect of advantage. If this be so in the adult, it is doubly so in the young subject, and any mistake in this respect is much more likely to be followed by injurious consequences in the latter than in the former. Now, the conditions which influence the effects of these agents are the state of the skin and the state of the nervous and vascular systems. With regard to the skin, it may be laid down as a general rule that when blisters are used as revulsives, the part to which they are applied should be as nearly as possible in a state of perfect health. With regard to the state of the system, this is even still more necessary to be enquired into; indeed, this all important if we hope to realize any of the expected benefits from these agents. Now, there are two states of the system almost equally unpropitious to their use, and these just the reverse of each other—the first is that in which high inflammatory excitement is present. That this is unfavorable to the beneficial operation of a blister as a revulsive is obvious, if we reflect for a moment upon the effects of this agent—these are local irritation and general excitement. Now, in all cases where an internal inflammation exists, the difficulty of resolving it by any means will be proportioned to the degree of general excitement accompanying it. If a blister be applied where this general excitement is already very great, one of the necessary consequences will be to augment this so greatly as to counteract in a greater or less degree, according to circumstances, the beneficial effects of the blister. Under this condition of things the internal inflammation will be aggravated instead of abated, in consequence of the increase of general excitement; hence the fact has been generally observed that if blisters are applied in the early periods of inflammatory complaints, or before suitable evacuations have been resorted to, they frequently do more harm than good—they merely add fuel to the fire. On the other hand, a state of great constitutional exhaustion and emaciation is also unfavorable to their operation; the reason here, however, is entirely different from that in the preceding case; the danger here is that from the impaired state of the vital energies the local inflammation of the blister may be followed by ulceration and gangrene. In the use of blisters, therefore, both these extremes should be carefully avoided. With regard to the condition most propitious, I think, to their use, it is that in which the general excitement is rather below than above the natural standard. When this is the case, there is less danger from any increase of excitement while the system is in the state most favorable to the transfer of irritations from one part to another. Now, if all this is applicable to the adult, we can easily see how much more so it must be in the case of the sensitive infant. In the use of blisters in children, especial reference should be had to the peculiarities of their temperament and constitution. This is more important, perhaps, than may at first sight appear. Every practitioner must have observed the extreme suffering which adults sometimes undergo from the irritation of blisters; in nervous and irritable habits I have seen a state of things thus induced little short of phrensy. In children of nervous temperament all this is much more likely to happen, and accordingly greater caution should be exercised.

In conclusion, if the foregoing statements be founded in truth, they would seem at once to expose the impropriety of the practice of resorting to the use of blisters on every trifling occasion. It cannot be said of them in truth, if they do no good they do no harm. If used ignorantly and indiscreetly, they are capable of doing much harm, even irreparable damage; but if used understandingly, with due regard to the conditions hinted at in this article, may often be made valuable auxilliaries in arresting and eradicating disease.*

* The foregoing paper discusses one side of an interesting topic. The question of counter-irritation has received considerable attention in the pages of this JOURNAL, and the Senior Editor has in several instances contributed articles denying its value in the majority, if not in all cases. He has yet to be convinced of the value of blisters, or any of the so-called counter-irritants, and especially so in children.

